# Complete Bilateral Ankle Fusion: A Rare Complication of Ankylosing Spondylitis

**DOI:** 10.7759/cureus.28094

**Published:** 2022-08-17

**Authors:** Zaid El-Fanek, Tetyana Gorbachova, Irene Tan

**Affiliations:** 1 Rheumatology, Philadelphia College of Osteopathic Medicine, Philadelphia, USA; 2 Radiology, Einstein Medical Center Philadelphia, Philadelphia, USA; 3 Rheumatology, Einstein Medical Center Philadelphia, Philadelphia, USA

**Keywords:** case report, ankylosis, dual joint fusion, spondyloarthropathies, ankle ankylosis, ankle joint fusion, ankylosing spondylitis

## Abstract

Ankylosing spondylitis (AS) is a chronic inflammatory disorder primarily affecting the axial skeleton and is strongly associated with a positive human leukocyte antigen B27 (HLA-B27) genotype. Patients typically present with chronic low back pain that typically starts before the age of 40 years. Common initial clinical features include lower back, hip, and joint pain with stiffness that is worse in the morning and with inactivity. As the disease progresses over a prolonged period, it leads to fusion of sacroiliac joints and ankylosis of the vertebrae with the iconic "bamboo spine" on imaging. Joint fusion or ankylosis is the sequela of either undiagnosed or untreated AS. We report a case of a 69-year-old male with complete fusion of the ankle joint, hindfoot, and midfoot of both feet in the clinical context of an incidental finding of an ankylosed spine on computed tomography (CT) imaging. The ankle joint is a very uncommon site for fusion in ankylosing spondylitis. We would like to suggest the terminology “boot sign” for this rare radiographic finding of complete ankle and subtalar fusion given the appearance of a boot. “Boot sign” is associated with either inability to ambulate or a steppage gait from loss of ankle dorsiflexion as a result of ankle and hindfoot fusion with or without fusion of forefoot and midfoot.

## Introduction

Ankylosing spondylitis (AS) is a chronic immune-mediated, inflammatory arthritis classically affecting the axial skeleton. Sacroiliac joints and apophyseal, discovertebral and costovertebral articulations of the spine are typically affected. Extra-axial manifestations may also include peripheral arthritis, uveitis, inflammatory bowel disease, and psoriasis [1,2]. In North America, there is an estimated prevalence of 31.9 per 10,000, with a higher prevalence associated with individuals of a lower socioeconomic background, who are also more prone to develop worse functional outcomes due to the disease [3]. The combination of osteitis and productive bone changes leads to syndesmophyte formation across the intervertebral discs, that over a prolonged duration results in the classic sign of a "bamboo" spine on radiographs. Pathologic involvement of the spine leads to clinical manifestation of chronic low back pain of insidious onset before age 40, typically in the second to third decade of life, with a modest male predominance [1-2,4]. Unfortunately, the insidious nature of the disease can lead to delays of up to eight to 10 years from the clinical presentation of symptoms to an official diagnosis [1]. Thus, utilizing and developing classification or diagnostic criteria is essential to establish early diagnosis and prevent disease progression.

Of the multiple proposed criteria, the most commonly used ones are the modified New York Classification Criteria and the modified Berlin Algorithm [5,6]. According to the former, AS is diagnosed if there is evidence of sacroiliitis in addition to two of the three following clinical criteria: a minimum of three months of inflammatory low back pain, limited range of motion of the lumbar spine, and limited chest expansion. In addition, patients are positive for sacroiliitis when there are severe erosions with partial fusion of one sacroiliac joint or sclerosis with some erosions present in both sacroiliac joints [5].

The modified Berlin algorithm proposes that evidence of sacroiliitis on plain radiographs is sufficient for diagnosis as long as the patient also has at least three months of chronic inflammatory back pain starting before age 45 and at least one associated clinical feature. These features include heel pain or enthesitis, asymmetric arthritis, a positive family history of AS, alternating buttock pain, inflammatory bowel disease, psoriasis, uveitis, dactylitis, a good response to non-steroidal anti-inflammatory drugs, or elevated acute phase reactants like the erythrocyte sedimentation rate (ESR) or C-reactive protein (CRP). If no radiographic evidence of sacroiliitis is established, patients must meet four of the eleven associated clinical features or two to three of these features in addition to a positive human leukocyte antigen B27 (HLA-B27) genotype. If suspicion remains high despite negative HLA-B27 testing, magnetic resonance imaging (MRI) is recommended to evaluate sacroiliitis [6].

Due to its high association with AS, with a greater than 90% prevalence in Caucasians and 50% in African Americans, genetic testing for this marker can be used to support the diagnosis. Rheumatoid factor and antinuclear antibodies are frequently negative in AS. Laboratory testing for acute phase reactants measuring ESR and CRP is not considered diagnostic of the disease since these markers can be normal in some patients [7]. However, certain studies have found elevated CRP levels in 40% of patients with AS [8], so these markers remain helpful in establishing a diagnosis. However, despite these established criteria and the various recommended markers, there is still potential for insufficient disease recognition and delay in diagnosis from the onset of symptoms, contributing to poorer functional outcomes and patient quality of life.

## Case presentation

**Figure 1:**
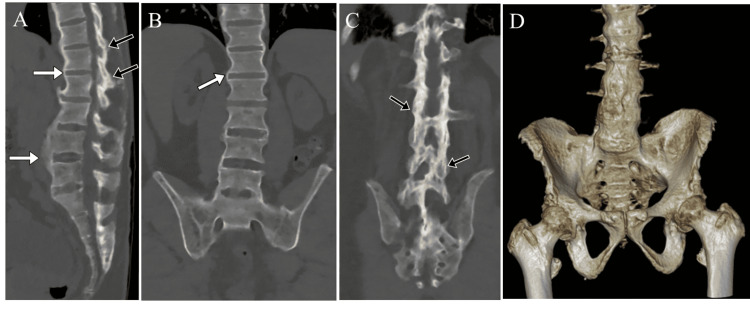
CT of the abdomen and pelvis. Sagittal (A) and coronal (B, C) reformatted images demonstrate findings of advanced ankylosing spondylitis, further illustrated by coronal three-dimensional volume-rendered CT image (D). Note extensive syndesmophyte formation throughout the spine (white arrows), fusion of the posterior elements (black arrow) and bilateral fusion of the sacroiliac joints.

A 69-year-old African American male with a past medical history of chronic low back pain and bilateral lower extremity swelling of unknown duration, ambulatory dysfunction with inability to ambulate for nearly two decades, gout, and chronic kidney disease presented to his primary care physician complaining of bilateral flank pain with associated nausea and vomiting. He was sent to the emergency department to be evaluated for acute worsening of renal function with serum creatinine of 14.9 mg/dL from last known serum creatinine of 2.6 mg/dL eight years ago. Additional laboratory tests in the emergency department revealed a glomerular filtration rate (GFR) of 3 mL/min, blood urea nitrogen (BUN) of 137 mg/dL, potassium of 6.1 mmol/L, and bicarbonate levels of 14 mEQ/L. The HLA-B27 status of the patient was unknown with no significant family history. Vital signs showed an elevated blood pressure of 182/68 mmHg but were otherwise within normal range. At initial evaluation, physical examination was negative for any skin lesion or rash, joint pain or swelling, and negative for extraarticular manifestations of AS. The patient was admitted for the initiation of hemodialysis. Urinalysis showed moderate blood, so a computed tomography (CT) scan of the abdomen and pelvis was ordered to investigate for possible kidney calculi. The CT scan was negative for acute abnormalities but incidentally revealed radiographic evidence of advanced ankylosing spondylitis (Figure 1).

**Figure 2:**
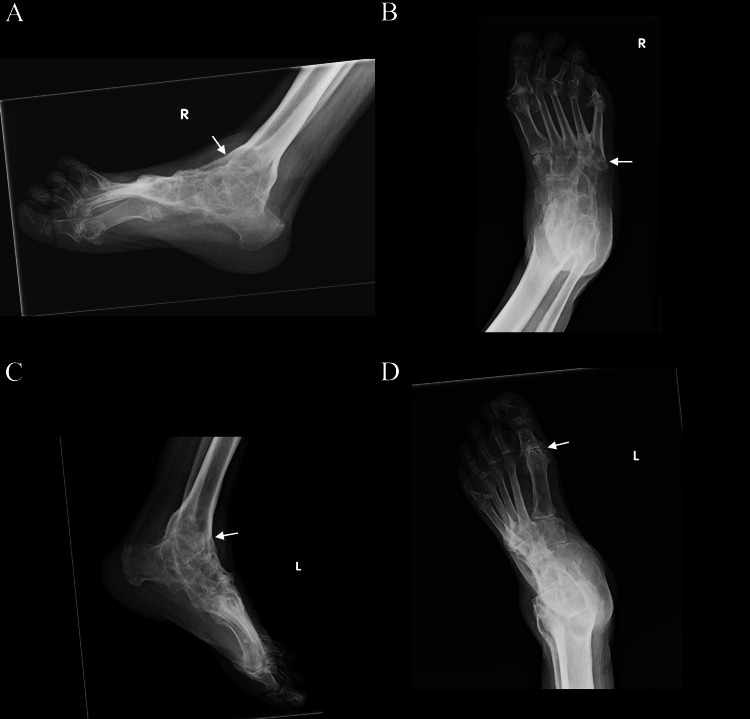
Radiographs of the feet during most recent hospital admission. Lateral views of the right (A) and left (C) foot demonstrate chronic osseous fusion of the right and left ankle joints, posterior subtalar joints, and the transverse tarsal joints. Anteroposterior views of the right (B) and left (D) feet demonstrate erosive changes along the metatarsophalangeal joints, more severe in the right foot compared to the left, and fusion of the second through fifth tarsometatarsal joints of the right foot.

On the third day of hospitalization, the patient complained of acute bilateral hand and foot pain typical of his acute gout flare in the setting of worsening kidney disease. The patient endorsed drinking beer as well as eating red meat and shellfish prior to hospitalization. On physical examination, he was found to have warm, boggy, tender swelling of bilateral second metacarpophalangeal joints, the knees, and the metatarsophalangeal joints of all digits on both feet. His ankles had no range of motion. In addition, a very large tophus was noted on the right elbow. He was diagnosed with acute, polyarticular, tophaceous gout with a serum uric acid level of 8.9 mg/dL. Allopurinol 50 mg daily was initiated, increasing to 100 mg daily following the first day, in addition to intravenous methylprednisolone 70 mg daily for his gout flare. X-rays of the hands and feet were ordered to assess the extent of damage from gouty erosions. Radiographs of the feet demonstrated erosions along the metatarsophalangeal joints while also revealing complete ankylosis and fusion of the ankle joint, hindfoot, and midfoot bilaterally, and the tarsometatarsal joints on the right (Figure 2).

**Figure 3:**
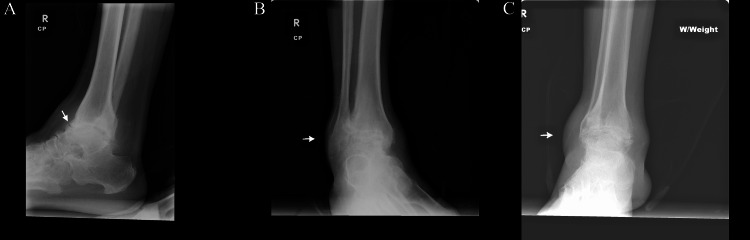
Radiographs of the right ankle 16 years before most recent hospital admission. Lateral view (A), mortise view (B), and anteroposterior view (C) with weight bearing demonstrate marked soft tissue swelling over the ankle, osteophytes, uniform joint space narrowing of the ankle and subtalar joints, and partial fusion of the right ankle joint as well as small calcaneal enthesophytes at the insertion of the Achilles tendon.

The patient's acute polyarthritis, including foot pain, resolved on his fourth day of treatment. However, there was still no range of motion in his ankles despite significant improvement in all the other joints. At this time, he reported he had not been able to ambulate normally for around two decades. Further review of past imaging corroborated this fact. An X-ray of the right ankle done 16 years ago showed significant soft tissue swelling around the ankle joint and uniform joint space narrowing with partial fusion of the ankle joints. Osseous ankylosis was less prominent when compared to recent imaging during this hospitalization (Figure 3). Although radiographs from 16 years ago were available for comparison, a hard copy of the medical record of that clinical encounter was not available for review. It is unknown if diagnosis and treatment for inflammatory arthritis was offered to him at the time.

## Discussion

We present a rare case of ankylosing spondylitis (AS) with the fusion of both ankles that was either unrecognized, untreated, or a combination of both for multiple decades. In addition, the patient himself is unaware that his joint condition was treatable. AS in this case has features of both axial and peripheral joint involvement demonstrating complete bilateral ankle fusion and hindfoot and midfoot fusion as a late-stage, rare complication of the disease. We get a remarkable glimpse of the radiographic disease progression from partial fusion to complete fusion over a 16-year period without pharmacologic intervention (Figure 2 vs Figure 3). We would like to suggest the terminology “boot sign” for Figures 2A, 2C of the right and the left ankle, respectively. The “boot sign” of the ankle on radiographs depicts ankle fusion and subtalar fusion or ankylosis with the complete loss of ankle and hindfoot mobility akin to the appearance of a boot. This is regardless of whether the midfoot or forefoot is involved. The analogy was made and the term “boot sign” was coined by Irene J. Tan, MD. Clinically, the “boot sign” would be associated with gait disturbance, such as steppage gait, or loss of ability to ambulate from a complete loss of ankle and subtalar range of motion. There are multiple examples of radiological signs that are used by different radiology subspecialties, sometimes within the same subspecialty, while referring to the different pathologies in different organ systems. Examples include “crescent sign,” “snowman sign,” and “ghost sign.” It is therefore essential that each sign is quoted in a specific clinical context. The value of an imaging sign is in its quick visual association that conveys several imaging features at once and in many cases points to the diagnosis. To that extent we believe that “boot sign” in the case presented will serve such a purpose. We believe that the “boot sign” of the ankles with its implication for gait dysfunction would be an analogy easily appreciated by the bone radiologists and all care providers, and not easily confused with “boot-shaped heart” on chest radiographs seen in patients with tetralogy of Fallot. 

Foot involvement is a relatively common occurrence in AS. However, there is limited data on ankylosis of the foot, and a paucity of ankylosis of the ankle joint in medical literature. When there is foot involvement in AS, a commonly affected area is the calcaneus in the hindfoot, with key areas of involvement at the posterior surface above the Achilles tendon attachment or the plantar surface anterior to the plantar fascia insertion [9]. This usually presents as focal erosions at the attachment of the Achilles tendon or with plantar calcaneal enthesophytes and plantar fasciitis [9,10]. This last feature was also seen in our case with the X-ray of the right ankle from 16 years ago showing a calcaneal spur and small enthesophyte at the insertion of the Achilles tendon (Figure 3A).

Other studies have shown that the midfoot is another area that is also frequently involved in AS, typically presenting as tarsitis and resulting in structural damage and impairment [10]. This inflammation of the tarsal joints has the potential for permanent changes that can substantially worsen patient outcomes. Some studies have shown that a form of tarsal fusion similar to that at the spine in AS can occur [9], especially at the intertarsal joints [9,10]. There are other specific instances depicting this tarsal fusion. However, many are limited to cases of juvenile spondyloarthropathy with ankylosis of the foot limited to either the intertarsal or tarsometatarsal segments and do not include ankylosis at the ankle joint. In addition, multiple tarsal joint fusion is considered uncommon. There was one case presented with bilateral tarsal ankylosis with multiple joint involvements [11], as well as another similar case with radiographs showing complete fusion of all tarsal joints with known juvenile spondyloarthropathy associated with Crohn's disease [12]. In our case, this tarsal fusion was also present, as seen in the more recent X-ray imaging (Figure 2A). This particular fusion was not evident in the previous imaging from 16 years ago (Figure 3A), likely depicting the progression of the partial to complete fusions of the disease.

Similarly, a study using magnetic resonance imaging (MRI) to view soft tissue, cartilage, and bone alterations in the feet of patients with AS showed that 69% had midfoot involvement, 22% had ankle involvement, and 9% of the patients presented with ankylosis in the midfoot. Although there was joint space narrowing in 5% of this cohort at the ankle, no ankylosis was noted at the ankle [10]. However, it is important to note that this study was also limited by a small sample size of 42 feet across 21 patients.

It is also important to note that several clinical conditions may lead to ankle fusion and should be considered in the differential diagnosis. Such conditions include posttraumatic or postinfectious ankylosis, and end stage arthropathy resulting from various disorders such as osteoarthritis, chronic rheumatoid arthritis or chronic gout. It is the extent of osseous ankylosis across the ankle and hindfoot along with its uniformity and bilaterality that may help differentiate AS as the most likely cause. Larger studies are needed to find the true prevalence of ankle joint ankylosis in AS and to further understand the possible interplay between diseases such as gout and ankylosing spondylitis in a setting of ankle joint fusion.

## Conclusions

There are limited reports of ankylosis of the foot in patients with AS, most of which focused on asymmetric ankylosis of the midfoot at the tarsal joints. In this case, we report a rare presentation of bilateral ankle joint ankylosis in AS as an incidental finding while searching for gouty erosions during an acute gout flare. This is in the setting of an additional incidental finding of an ankylosed spine during workup for kidney failure. Early detection of and intervention against ankylosing spondylitis are necessary for better patient outcomes and quality of life. Peripheral arthritic manifestations such as enthesitis of the lower extremities, leading to pain and swelling of the ankles, may indicate unrecognized AS, especially in patients complaining of chronic back pain. However, it is unknown if our patient complained of chronic back pain or had ongoing AS at the time of initial radiographic imaging. Regardless, early diagnosis or recognition of midfoot involvement is equally crucial since long-term tarsitis has the potential to permanently damage the structure and lead to poor functional outcomes through tarsal fusion. We suggest the terminology “boot sign” for radiographic findings of complete ankle fusion and hindfoot fusion akin to the appearance of a boot. It is important to note that the multiple superimposed issues can potentially complicate the clinical picture in our case.
